# Tailoring Microstructure and Properties of W-Mo-Cu Composites Fabricated via Infiltration Sintering: Effects of Graphene Addition and Skeleton Relative Density

**DOI:** 10.3390/ma18112539

**Published:** 2025-05-28

**Authors:** Jinwen Cai, Qiaoling Jiang, Keqin Feng, Hongling Zhou

**Affiliations:** 1 School of Mechanical Engineering, Sichuan University, Chengdu 610065, China; caijinwen@stu.scu.edu.cn (J.C.); jiangqiaoling@stu.scu.edu.cn (Q.J.); 2 College of Materials Science and Engineering, Chongqing University, Chongqing 400044, China

**Keywords:** graphene, infiltration sintering, W-Mo-Cu composites, densification, properties

## Abstract

W-Mo-Cu composites show promise for advanced applications, but their properties require optimization. In this study, a novel approach utilizing Cu-coated graphene (Cu@Gr) reinforcement with skeleton relative density adjustment was employed to tailor the microstructure and properties of W-Mo-Cu composites fabricated via infiltration sintering (1300 °C, 1.5 h). The results revealed that Cu@Gr significantly promoted sintering densification, modified the phase composition, and enhanced the properties of the composite. Specifically, the addition of 0.4 wt.% Cu@Gr resulted in a relative density of 98% for the composite, representing an 8% increase compared to the material without Cu@Gr. Furthermore, when higher amounts of Cu@Gr were incorporated, the composite consistently exhibited a high degree of densification. In addition to the primary W, Mo, and Cu phases, molybdenum carbide, Mo_2_C, was formed at 0.4 wt.% Cu@Gr, with its content rising proportionally to graphene dosage. Notably, the composite containing 0.6 wt.% Cu@Gr exhibits the highest thermal conductivity and electrical conductivity, showing 64% and 73% increases, respectively, versus Cu@Gr-free samples. Additionally, although W-Mo green compact density variations (73–85%) did not compromise graphene-induced densification, a higher green compact density reduced the thermal/electrical conductivities but increased the hardness. These findings demonstrate that controlled Cu@Gr incorporation and green compact optimization synergistically improve the properties of W-Mo-Cu composites, providing insights into high-performance material design.

## 1. Introduction

Refractory metals, such as tungsten (W) and molybdenum (Mo), possess high melting temperatures, low thermal expansion coefficients, good ablative resistance, and high hardness. These properties make them instrumental in the fabrication of structural materials used in aerospace applications and nuclear reactors [1,2]. In contrast, as a low-melting-point metal, copper (Cu) not only has excellent thermal conductivity and electrical conductivities but also exhibits good toughness [3,4]. Thus, W-Mo-Cu composite, a novel and promising pseudo-alloy with a tailorable composition ratio integrates the advantageous physical properties of metal W, Mo, and Cu, which can be widely applied in various fields, including arcing electrodes, electronic packaging materials, high-voltage electrical contacts, sweating materials, and electromagnetic gun-rail materials [5,6,7]. Nevertheless, the fabrication of W-Mo-Cu composites presents challenges due to the significant differences in the melting points of the constituent elements (W: 3410 °C, Mo: 2623 °C, and Cu: 1083 °C), as well as the insolubility of W and Cu, and Mo and Cu [8,9]. Currently, the fabrication of W-Mo-Cu composites predominantly relies on powder metallurgy methods, including liquid-phase sintering, field-assisted sintering techniques, and infiltration sintering [7,10,11]. The liquid-phase sintering method is simple and controllable but requires high temperatures (1100–1400 °C) and long times (3–4 h), which may form Cu melt pools and adversely affect the composite’s microstructure. Furthermore, although the pretreatment of powders can enhance the properties of W-Mo-Cu composites, it also increases the preparation time [12]. Field-assisted sintering methods, such as spark plasma sintering, microwave sintering, and large current electric field sintering, utilize external physical fields to activate the sintering process, thereby reducing the temperature (800–1000 °C) and preparation time (5–10 min) for composites. However, the resulting W-Mo-Cu composites exhibit suboptimal ies with a maximum relative density of approximately 94% [10,11,13]. Although it requires similarly high sintering temperatures (1200–1400 °C) and extended durations (4 h) as liquid-phase sintering, the infiltration sintering method produces composites with significantly reduced porosity by effectively filling the W or Mo skeleton with Cu. This enables the preparation of high-density W-Mo-Cu composites without powder pretreatment [14,15]. However, the high temperature and long duration of the infiltration sintering process still pose challenges, such as Cu phase volatilization, weakened interfacial bonding, and grain coarsening during sintering, which may adversely affect the properties of W-Mo-Cu composites. To address these challenges, elements such as Co, Ni, and Fe are frequently added as sintering activators in pseudo-alloys, reducing the activation energy and sintering temperature while enhancing the overall performance [16,17,18]. However, these elements can negatively affect the electrical conductivity and thermal conductivities of pseudo-alloys, which undoubtedly impacts their practical applications [19]. Therefore, it is essential to select new additives that do not compromise the excellent electrical and thermal conductivities of W-Mo-Cu composites.

Graphene, which is renowned for its exceptional electron mobility (2× 10^5^ cm^2^/(V·S)), high carrier density (10^13^ cm^−2^), ultrahigh thermal conductivity (up to 5300 W/(m·K)), and remarkable mechanical strength (130 GPa), has been successfully integrated into composite materials such as aluminum-, magnesium-, copper-, and titanium-based systems. For instance, Hu et al. [20] fabricated graphene-aluminum matrix composites via electromagnetic stirring and casting processes, achieving optimal performance at 0.2 wt. % graphene content with hardness, electrical conductivity, and thermal conductivity increased by 42%, 5%, and 38%, respectively, compared to pure aluminum. Furthermore, Zhao et al. [21] and Chen et al. [22] demonstrated that graphene incorporation facilitates grain refinement while significantly enhancing the mechanical, electrical, and thermal properties of metal matrices. However, when graphene is incorporated into metal matrix composites, it still encounters challenges such as difficulties in dispersion, a tendency to agglomerate, and susceptibility to reactions with the matrix [23,24,25,26]. Consequently, surface pretreatment of graphene is often required, with the electroless deposition of a metal layer onto its surface being a relatively effective modification technique[21,23,27,28].

In this study, surface-modified graphene (Cu@Gr) was fabricated via electroless plating and subsequently incorporated into a W-Mo-Cu composite matrix with varying Cu@Gr contents (0.2–1.0 wt. %) to synthesize Cu@Gr-reinforced W-Mo-Cu (Cu@Gr/W-Mo-Cu) composites through infiltration sintering. The effects of Cu@Gr addition on the microstructure and properties of the composites were systematically investigated. Furthermore, considering the critical role of the relative density in W-Mo green compacts for successful infiltration, a complementary study was conducted to evaluate how varying relative densities (73–85%) of the W-Mo skeleton, combined with the optimized Cu@Gr content, influence the final composite performance. This dual-parameter optimization strategy provides insights into both the compositional and structural controls for enhancing W-Mo-Cu composites.

## 2. Experimental

### 2.1. Materials

**Figure 1:**
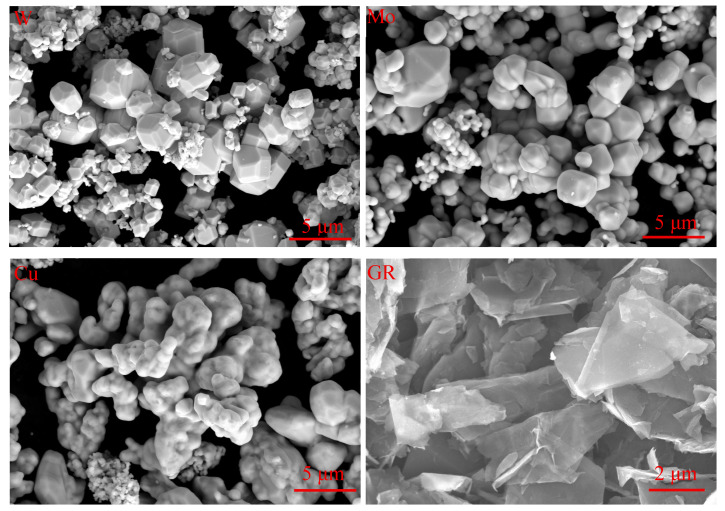
Microstructure of the raw material powders.

W powders (99.9% purity; average particle size between 1–3 μm), Mo powders (99.9% purity; average particle size between 1–3 μm, Xiamen Golden Egret Special Alloy Co., Ltd. (Xiamen, China)), Cu powders (99.9% purity; average particle size of 5 μm, Zhongye Xindun Co., Ltd. (Xingtai, China)) and Graphene powders with 5–10 layers (99.0% purity; average particle diameter of 5 μm, Shenzhen Suiheng Graphene Technology Co., Ltd. (Shenzhen, China)) were used as raw powders for the experiment. Figure 1 shows the SEM micrographs of the raw powders used for the composite fabrication. The W powder particles exhibited irregular polyhedral morphologies with featureless surfaces, whereas the Mo powder consisted of irregular spherical particles. The Cu powder displayed ellipsoidal irregular shapes, with graphene presenting ultrathin flake-like structures. The auxiliary chemicals employed for graphene surface modification via electroless plating included (except for C_10_H_8_N_2_ and NaOH, all other raw materials were sourced from Chengdu Kelong Chemical Co., Ltd. (Chengdu, China)): polyvinyl alcohol (PVA), N-methylpyrrolidone (NMP), hydrochloric acid (HCl, 37%), stannous chloride dihydrate (SnCl_2_·2H_2_O), palladium chloride (PdCl_2_), copper sulfate pentahydrate (CuSO_4_·5H_2_O), potassium sodium tartrate tetrahydrate (KNaC_4_H_4_O_6_·4H_2_O), ethylenediaminetetraacetic acid disodium salt (Na_2_EDTA), sodium carbonate (Na_2_CO_3_), 2,2′-bipyridine (C_10_H_8_N_2_) (Chengdu Nuershi Technology Co., Ltd. (Chengdu, China)), sodium hydroxide (NaOH) (Chengdu Kelong Chemical Reagent Factory (Chengdu, China)), and formaldehyde (HCHO, 37%). All reagents were procured as analytical-grade materials and utilized without additional purification. Aqueous solutions were prepared using deionized water (resistivity > 18 MΩ·cm).

### 2.2. Graphene Surface Modification

**Figure 2:**
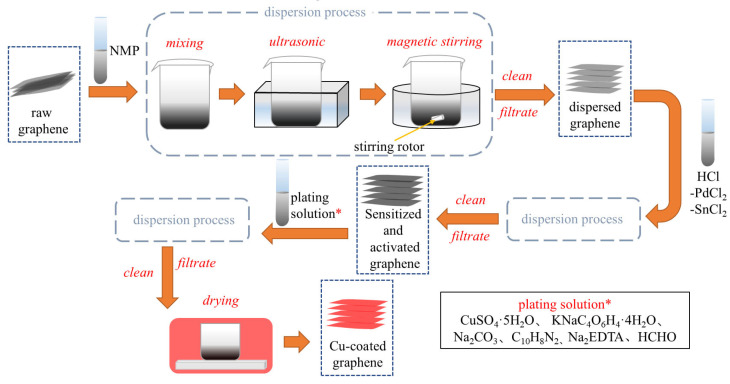
The schematic process of graphene surface modification [29].

**Figure 3:**
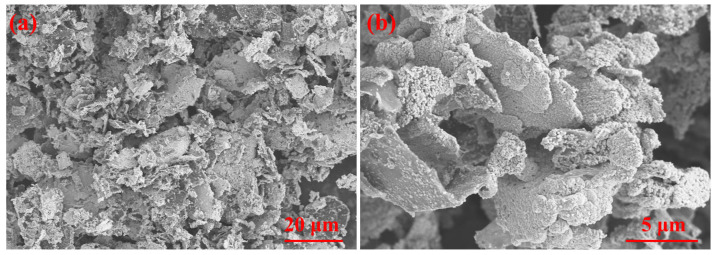
The microstructure of Cu@Gr: (**a**) 1000×, (**b**) 5000×.

Figure 2 illustrates the schematic process of graphene surface modification. Initially, graphene was treated with ultrasonication for 0.5 h and magnetic stirring for 1 h in an NMP solution to eliminate surface contaminants. The sensitization and activation solutions are then prepared by mixing deionized water, HCl, SnCl_2_·2H_2_O, and PdCl_2_ according to the formulation listed in Table 1. The dispersed graphene is immersed in this mixed solution and subjected to ultrasonication for 0.5 h followed by magnetic stirring for 1 h. The resulting material is subjected to vacuum filtration and rinsed with deionized water to yield sensitized and activated graphene. Next, the plating solution is formulated based on the composition specified in Table 2, with a pH adjustment to 12.5 using NaOH. Sensitized and activated graphene are introduced into a pH-controlled plating solution and processed under constant-temperature magnetic stirring in a water bath. The final product is isolated through filtration, thoroughly washed with deionized water, and vacuum-dried at 60 °C for 4 h to obtain Cu@Gr. As revealed by the microstructure analysis in Figure 3, the modified Cu@Gr exhibits a continuous and conformal coating across the majority of graphene surfaces.

### 2.3. Preparation of Cu@Gr-Reinforced W-Mo-Cu Composites

This study primarily investigated the 40 wt.% W-40 wt.% Mo-20 wt.% Cu system. W and Mo powders were weighed to 2 wt.% pre-added Cu powder to enhance melt infiltration during subsequent sintering. Separately, 0.2 wt.%, 0.4 wt.%, 0.6 wt.%, 0.8 wt.%, and 1.0 wt.% Cu@Gr powder was systematically introduced to the base mixture. The blended powders underwent mechanical alloying in a QM-BP planetary ball mill (ball-to-powder ratio of 3:1, Nanjing Nanda Instrument Co., Ltd., Nanjing, China) at 300 rpm for 3 h. The milled composite powder was then die-compacted using a DHM-YJB6 hydraulic press (Chengdu Dahua Hydraulic Machinery Factory, Chengdu, China) under 400 MPa uniaxial pressure with a 20 s dwell time, forming cylindrical green compacts (Ø13 mm × 11 mm height). The corresponding Cu compacts were pressed similarly for subsequent infiltration.

**Figure 4:**
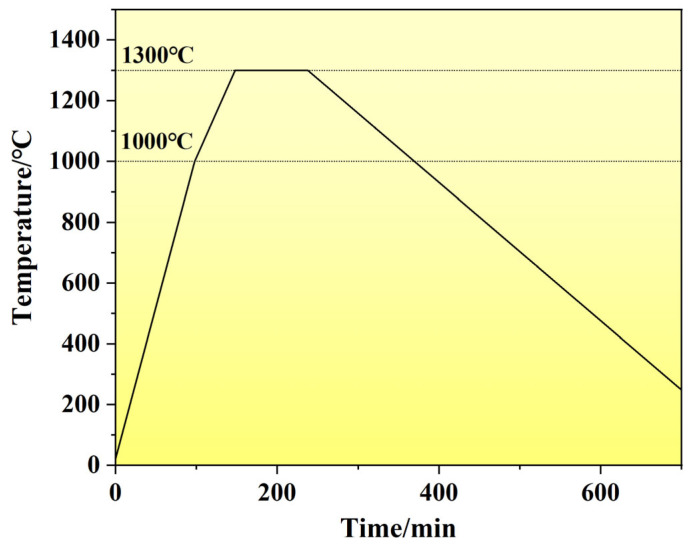
Sintering process of the infiltration sintering.

The W-Mo and Cu compacts were co-sintered in a ZT-40-21Y vacuum furnace (Shanghai Chenhua Science Technology Corp., Ltd., Shanghai, China) using a graphite crucible configuration, with the Cu block positioned above the compact. The furnace pressure was maintained below 2.2 × 10^−2^ Pa throughout the sintering process, as illustrated in Figure 4, which comprised four distinct phases: (1) rapid heating from ambient temperature to 1000 °C at 10 °C/min, (2) reduced-rate heating to 1300 °C at 6 °C/min, (3) 90-min isothermal holding at 1300 °C to complete Cu infiltration, and (4) furnace cooling to ambient temperature. Post-sintering, excess surface Cu was removed through mechanical machining to obtain the final composites.

### 2.4. Testing and Characterization

After sintering, the phases of Cu@Gr/W-Mo-Cu were analyzed using X-ray diffraction (XRD). The diffraction peaks in the XRD images were identified using the MDI Jade 6.0 software. The microstructures were examined using scanning electron microscopy (SEM). The elemental composition was analyzed using energy-dispersive X-ray spectroscopy (EDS). The density of the compact was measured using Archimedes’ method. The electrical conductivity, thermal conductivity, and microhardness of the compacts were assessed at room temperature (25 ± 1 °C) using a portable eddy current conductivity meter (FD102, Xiamen Fosite Electronic Technology Co., Ltd., Xiamen, China), laser thermal conductivity meter (LFA 457, NETZSCH–Gerätebau GmbH, Selb, Germany), and digital microhardness tester (HVD-1000AP, Shanghai Jujing Precision Instrument Manufacturing Co., Ltd., Shanghai, China), respectively. All reported data represent the averages of at least five test results.

## 3. Results and Discussion

### 3.1. Influence of Cu@Gr Contents on the Microstructure and Properties of Cu@Gr/W-Mo-Cu Composites

#### 3.1.1. Phases and Microstructures of W-Mo-Cu Composites with Different Cu@Gr Contents

**Figure 5:**
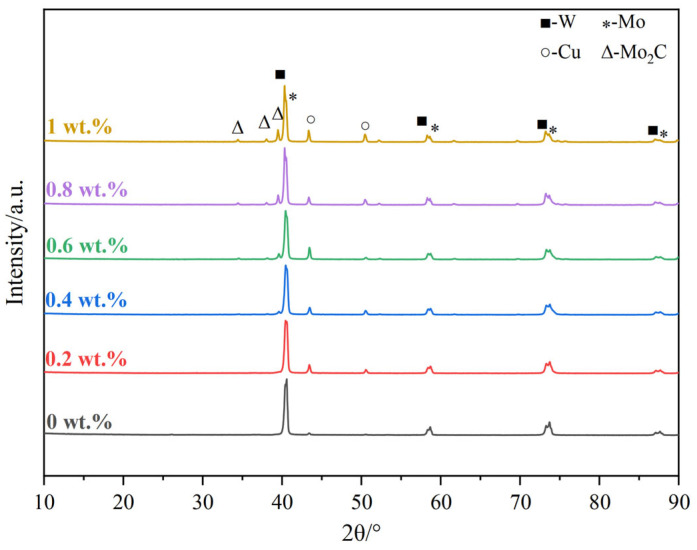
XRD patterns of W-Mo-Cu composites with different contents of Cu@Gr.

Figure 5 shows the XRD patterns of the W-Mo-Cu composites with different Cu@Gr contents. The data indicate that the W-Mo-Cu composite without Cu@Gr primarily consists of W, Mo, and Cu phases. Given that W and Mo belong to the same group of elements, they can form solid solutions with one another in the phase diagram. Consequently, W-Mo solid solutions are expected to form in the composite produced by infiltration sintering. Cao et al. observed W-Mo solid solutions dispersed at the W-Mo interfaces in W-Mo-Cu composites prepared via infiltration sintering at 1500 °C for 4 h, as evidenced by TEM [7,14]. However, the XRD patterns reveal that the diffraction peaks of W and Mo are distinct, indicating that no significant solid solution effect is present. This observation can be attributed to the relatively short sintering duration of only 1.5 h, which restricts the diffusion distance of W and Mo atoms, resulting in a limited degree of solid solution formation between W and Mo. Furthermore, the addition of 0.4 wt.% Cu@Gr, a small quantity of molybdenum carbide, Mo_2_C, begins to emerge in the W-Mo-Cu composite. As the Cu@Gr content increases, the intensity of the Mo_2_C diffraction peaks gradually increases, although the overall intensity of these peaks remains relatively low. Carbides, being hard phases, can enhance dispersion strengthening when they are uniformly distributed within the composites. However, the comparatively low conductivity of carbides may negatively impact the electrical conductivity of W-Mo-Cu composites. Therefore, to achieve W-Mo-Cu composites with optimal overall performance, the addition of Cu@Gr must be carefully regulated.

**Figure 6:**
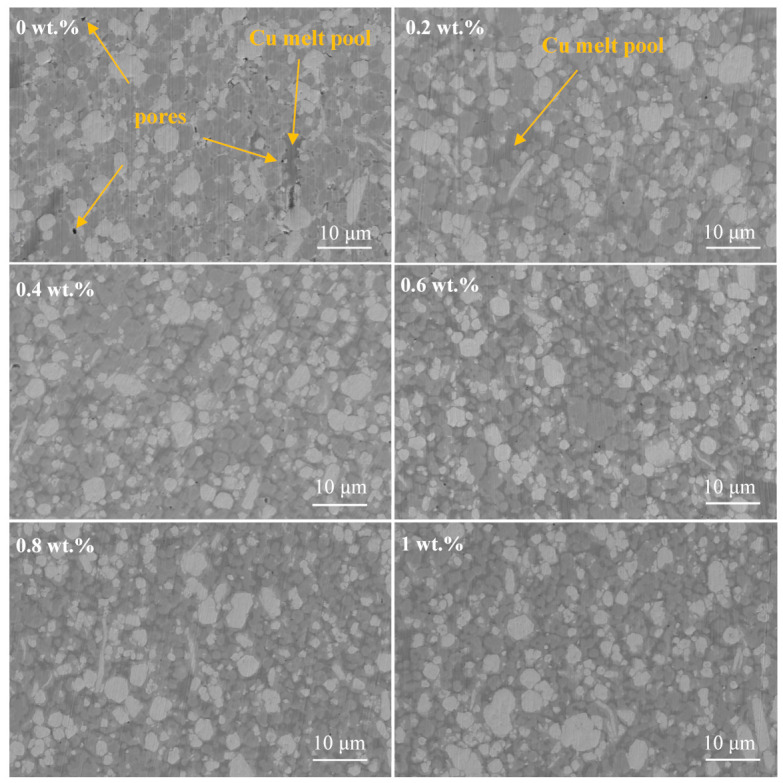
SEM images of W-Mo-Cu composites with different Cu@Gr contents.

**Figure 7:**
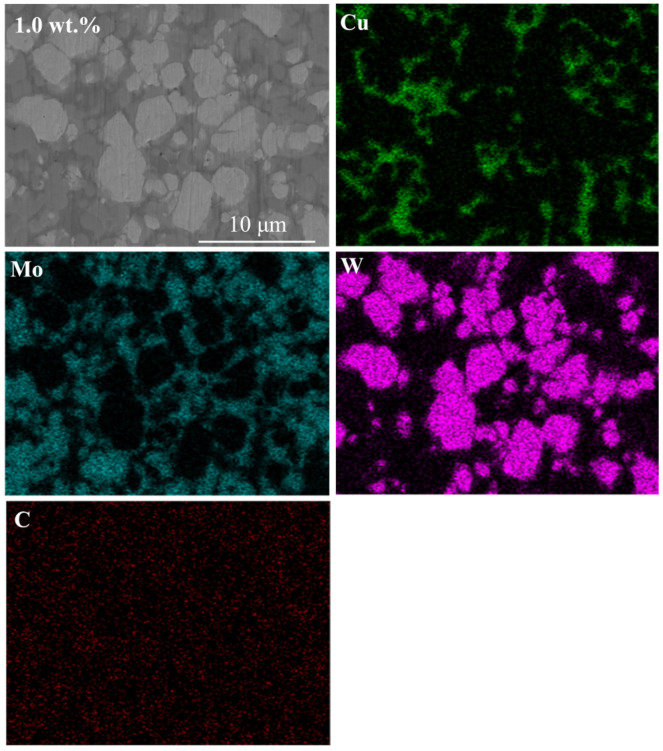
EDS analysis of the 1 wt.% Cu@Gr-added W-Mo-Cu composite.

Figure 6 shows the SEM images of the infiltration-sintered Cu@Gr/W-Mo-Cu composites. EDS analysis of the 1 wt.% Cu@Gr-added W-Mo-Cu composite was conducted, with results shown in Figure 7. EDS analysis reveals that the white areas in the SEM image correspond to the W phase, the light gray regions primarily represent the Mo phase, and the dark gray areas were attributed to the Cu phase. The W-Mo-Cu composite without Cu@Gr contains some circular pores of varying sizes, indicating insufficient infiltration of Cu into the W-Mo skeleton during processing, which resulted in incomplete filling of interparticle voids between W and Mo. Furthermore, the distribution of Cu is also not uniform, resulting in the formation of Cu melt pools. The addition of graphene leads to a gradual decrease in porosity and results in a more uniform microstructure, suggesting that graphene facilitates sintering densification. The addition of 0.2 wt.% graphene significantly enhances the density of the composite; however, minor pores remain, and a Cu melt pool is still present. Upon increasing the Cu@Gr addition to 0.4 wt.%, the microstructure of the composite material becomes notably denser and more uniform. The Cu melt pool no longer exists, and a uniform Cu network structure is formed. This improvement is attributed to the superior high-temperature wettability between graphene and Cu compared to that between W/Mo and Cu in the matrix [30,31]. The high wettability of graphene with Cu facilitates the flow of liquid Cu on the matrix surface contained with Cu@Gr. Although the direct wettability between Cu and W/Mo is limited, graphene serves as an intermediate layer, functioning as a “wetting bridge” that directs the liquid Cu to infiltrate the voids of the W-Mo skeleton along the graphene-covered pathway. Thus, Cu@Gr effectively contributes to sintering densification during infiltration.

From the element distribution in the EDS mapping, it can be observed that the distribution of each component is relatively uniform. The Mo phase serves as the matrix surrounding the W phase, while the Cu phase effectively fills the voids between the W and Mo phases, creating a uniform Cu network structure. However, the W phase exhibits slight particle coarsening, primarily due to the non-uniform particle size distribution of the raw W powder, which contains some larger particles. These larger particles hinder the rearrangement of particles during infiltration sintering, leading to the connection and coarsening of certain W phases [32]. Sintering necks are formed between the W, Mo, and Cu phases, tightly bonding them together and ultimately promoting the densification of the composites. Furthermore, the distribution of carbon is relatively uniform and not concentrated around the Mo phase, indicating that not all graphene has reacted to form carbides, and some free graphene still exists within the material.

#### 3.1.2. Properties of W-Mo-Cu Composites with Different Cu@Gr Contents

**Figure 8:**
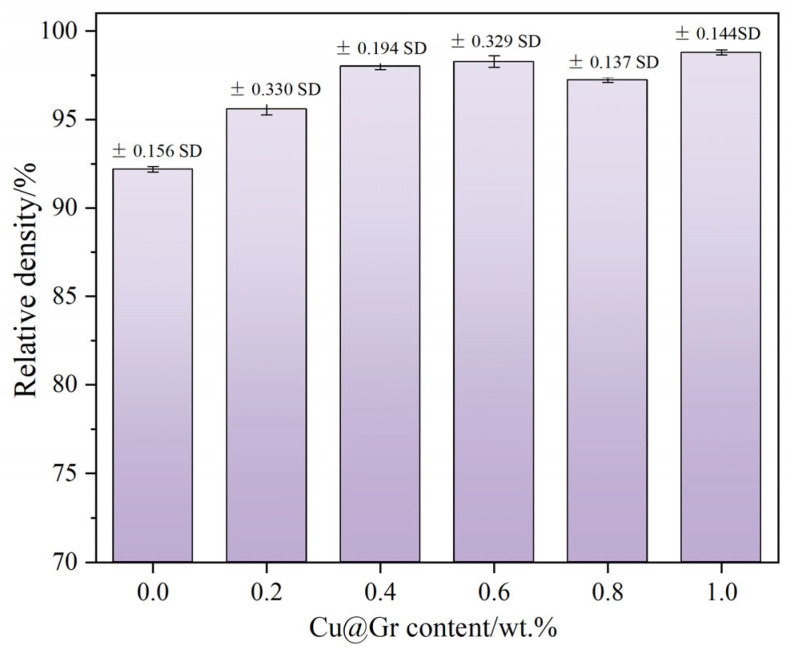
The relative density of W-Mo-Cu composites with varying contents of Cu@Gr.

Figure 8 illustrates the relative density of W-Mo-Cu composites with varying contents of Cu@Gr. The relative density of the composites significantly increases with the incorporation of Cu@Gr. This observation corresponds to the microstructural changes in the W-Mo-Cu composites depicted in Figure 6, where the addition of Cu@Gr progressively reduces both the number and size of pores within the composites. Notably, when Cu@Gr was not added, the wettability between Cu and W and Mo was poor, and Cu failed to adequately infiltrate into the W-Mo skeleton. The filling effect of copper in the pores between the W and Mo particles was mediocre, resulting in the lowest relative density. When the Cu@Gr content increases from 0 wt.% to 0.4 wt.%, the densification rate of the composites improves considerably; however, further increases in Cu@Gr content do not yield significant changes in the material’s densification, with values stabilizing around 98%. At this point, the densification of the composites improved by approximately 8% compared to that of the samples without Cu@Gr. In conclusion, the results indicate that Cu@Gr can effectively promote the sintering densification process of W-Mo-Cu composites during infiltration sintering, and a significant effect can be achieved when the Cu@Gr content reaches or exceeds 0.4 wt.%.

**Figure 9:**
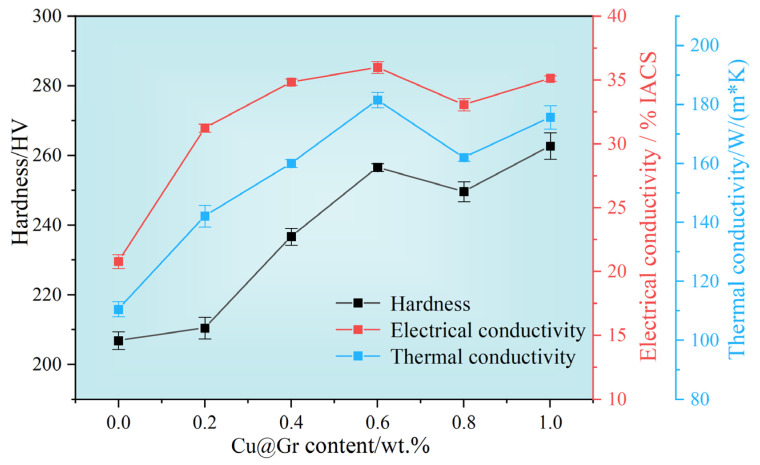
Thermal conductivity, electrical conductivity, and Vickers hardness of W-Mo-Cu composites with different contents of Cu@Gr.

Figure 9 shows the thermal conductivity, electrical conductivity, and Vickers hardness of W-Mo-Cu composites with varying Cu@Gr contents. As shown in the figure, both the thermal and electrical conductivities of the W-Mo-Cu composites are significantly enhanced, exhibiting similar trends as the Cu@Gr content increases. This is primarily attributed to the fact that the transfer of heat and electricity in metallic materials occurs mainly through the migration of free electrons. The presence of pores within the material can disrupt the continuity of the metal lattice, thereby increasing the resistance to the migration of free electrons. Therefore, the densification degree of the W-Mo-Cu composite is a crucial factor affecting its thermal and electrical conductivities. Furthermore, the thermal and electrical conductivities of W-Mo-Cu composites are influenced by the uniformity and structural integrity of the Cu network within the material, as the electrical and thermal conductivities of Cu are significantly higher than those of W and Mo [33,34]. Without the incorporation of Cu@Gr, Cu cannot sufficiently infiltrate the W-Mo skeleton, leading to a relatively low composite density and an incomplete and non-uniform Cu network, which adversely affects both the electrical and thermal conductivities. Conversely, the addition of Cu@Gr not only significantly enhances the composite’s density but also facilitates the formation of a more complete and uniform Cu network. Consequently, as the amount of Cu@Gr increases, the thermal and electrical conductivities of the W-Mo-Cu composites progressively improve.

Specifically, the W-Mo-Cu composite without Cu@Gr exhibits thermal and electrical conductivities of 110.61 W·m^−1^·K^−1^ and 20.81 %IACS, respectively. At a 0.6 wt.% addition of Cu@Gr, these values achieve the highest to 181.54 W·m^−1^·K^−1^ and 35.99 %IACS, reflecting approximate increases of 64% and 73% compared to the sample without any addition. However, when the Cu@Gr content exceeded 0.6 wt.%, the quantity of carbide produced during the sintering process rose, resulting in a gradual decline in both the thermal and electrical conductivities of the composite. Especially when the addition of Cu@Gr is 0.8 wt.%, the relative density of the composite decreases, resulting in a significant reduction in both the electrical and thermal conductivities of the composite at this composition. Nevertheless, due to the relatively low total content of carbides, this decrease remains relatively small.

Additionally, the hardness of the W-Mo-Cu composites gradually increases with the Cu@Gr contents. In graphene-reinforced composites, graphene serves as an efficient stress-transfer bridge. When a material is subjected to external loads, graphene can absorb and transfer a portion of the load, thereby mitigating the stress concentration within the metal matrix and enhancing the overall load-bearing capacity of the material, thus improving its strength [35,36]. Furthermore, the microstructure of the material is a critical factor influencing the hardness of the W-Mo-Cu composite. A higher relative density and fewer internal pores are conducive to an increase in material hardness. Moreover, the carbides formed during sintering can act as reinforcing phases, providing dispersion strengthening to the material. This reaction also promotes the formation of a strong interfacial bond between graphene and the W phase, effectively facilitating load transfer. Therefore, without the addition of Cu@Gr, the Vickers hardness of the material is 206.87 HV. As the Cu@Gr content in the composite increases to 0.6 wt.%, the hardness significantly improves, reaching 256.57 HV, which represents a 24% increase compared to the absence of Cu@Gr. However, as the Cu@Gr content continues to increase, the change in material hardness becomes marginal, with a slight decrease due to the reduction in the relative density of the composite at a Cu@Gr content of 0.8 wt. % and with a maximum hardness of 262.7 HV achieved at a Cu@Gr content of 1 wt.%.

In conclusion, the addition of 0.6 wt.% Cu@Gr results in the W-Mo-Cu composite exhibiting a higher degree of densification, a highly uniform microstructure, and optimal electrical and thermal conductivities, thereby establishing it as the optimal addition.

### 3.2. Influence of W-Mo Green Compact Relative Density on the Microstructure and Properties of Cu@Gr/W-Mo-Cu Composites

#### 3.2.1. Phases and Microstructures of W-Mo-Cu Composites with Varying Relative Densities of W-Mo Green Compacts

Given that the relative density of the W-Mo green compact significantly influences the microstructure and properties of the composite fabricated via infiltration sintering, this section maintains a constant Cu@Gr addition of 0.6 wt. % in W-Mo-Cu composites and investigates the effect of the relative density of W-Mo green compact on the microstructure and properties of the composites.

**Figure 10:**
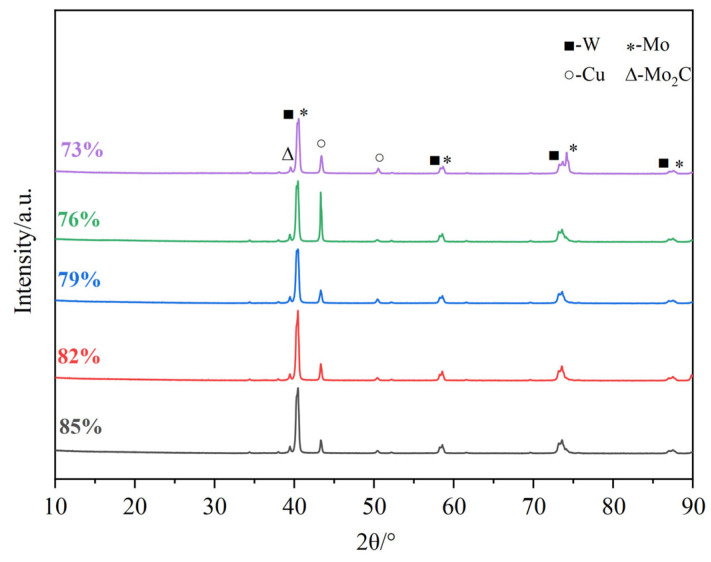
XRD patterns of W-Mo-Cu composites with varying relative densities of W-Mo green compacts.

Figure 10 shows the XRD patterns of W-Mo-Cu composites with varying relative densities of W-Mo green compacts. As shown, the phase composition of the W-Mo-Cu composites remains unchanged with variations in the relative density, consisting of the W, Mo, Cu, and Mo_2_C phases. The peaks corresponding to the Mo_2_C phase remain largely consistent, suggesting that the relative density of the W-Mo green compact had a minimal impact on both the phase composition and carbide formation reaction within the composites.

**Figure 11:**
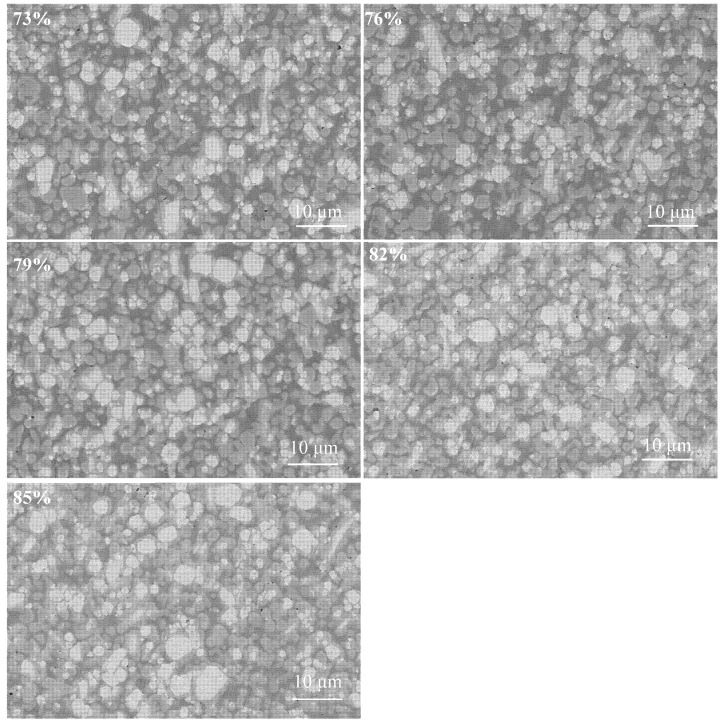
SEM images of W-Mo-Cu composites with varying relative densities of W-Mo green compacts.

Figure 11 shows the SEM images of W-Mo-Cu composites with varying relative densities of W-Mo green compacts. As shown, the five groups of samples exhibit relatively uniform and dense structures, with the Cu phase well infiltrating the skeleton. Furthermore, as the relative density of the W-Mo green compacts decreases, the dark gray Cu phase gradually increases. The variation in the relative densities of the W-Mo green compacts does not influence the promoting effect of Cu@Gr on the densification of the composites. Generally, a higher relative density of the W-Mo green compacts correlates with reduced porosity and pore size, which results in a greater capillary force exerted on the Cu melt during sintering, thereby enhancing its infiltration capability. Conversely, the presence of larger pores in the matrix at lower green compact densities can impede the flow and infiltration of the Cu melt. In this study, due to the favorable wettability between graphene and Cu at elevated temperatures, the densification of the composite remained unaffected by the decrease in the relative density of the W-Mo green compact. Instead, as the porosity of the green compact increased, the volume of Cu that could infiltrate also increased. The greater the amount of liquid Cu, the more it facilitated the rearrangement and redistribution of W-Mo particles, thereby promoting the sintering densification of the composite [37].

#### 3.2.2. Properties of W-Mo-Cu Composites with Varying Relative Densities of W-Mo Green Compacts

**Figure 12:**
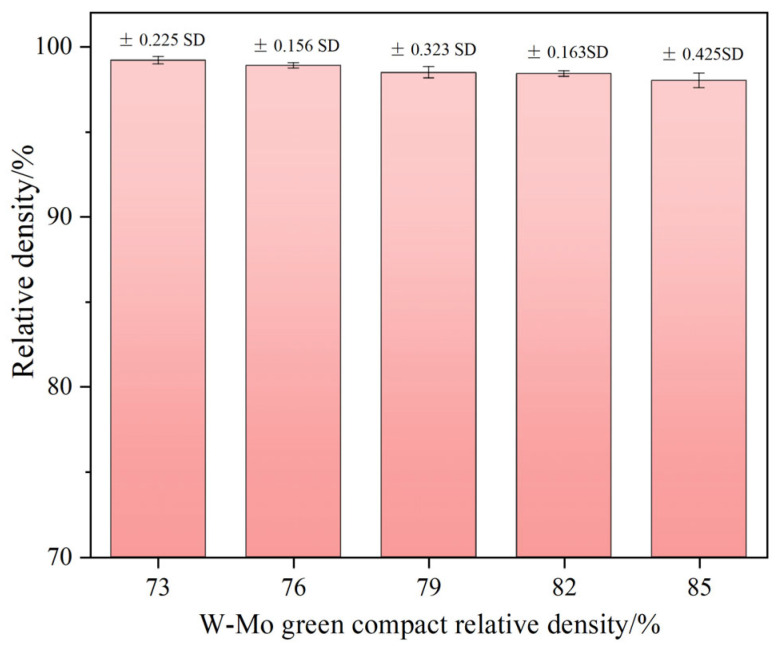
The relative density of W-Mo-Cu composites with varying relative densities of W-Mo green compacts.

Figure 12 illustrates the relative density of W-Mo-Cu composites with varying relative densities of the W-Mo green compacts. It is evident that as the relative density of the W-Mo green compacts decreases, the relative density of the composites gradually increases, reaching a maximum value of 99.2% when the relative density of the green compacts is 73%. Analysis of the SEM images of the W-Mo-Cu composites presented in Figure 11 shows that, despite variations in the relative density of the W-Mo green compacts, the samples consistently exhibit a high degree of densification with minimal differences. Thus, a lower relative density of the W-Mo green compact correlates with a greater variation in the densification of the composites. Specifically, when the relative density of the green compact is 73%, the final density of the sample increases by 35.9%, while at a relative density of 85%, the final density increases by 15.3%. This indicates that the relative density of the W-Mo green compact significantly influences the densification process of the composite material.

**Figure 13:**
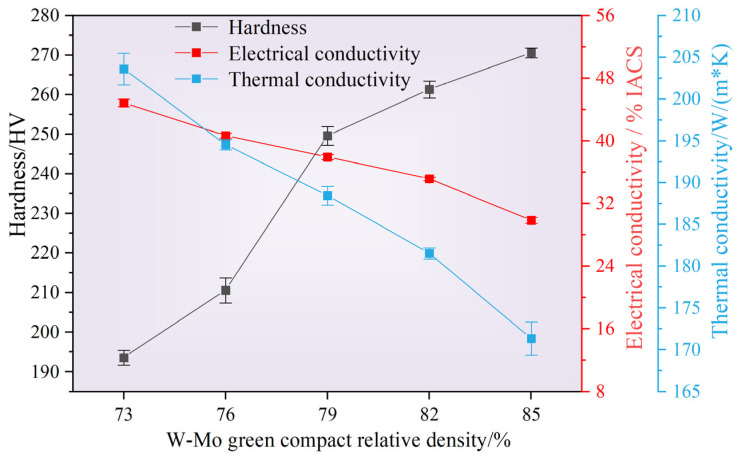
Thermal conductivity, electrical conductivity, and Vickers hardness of W-Mo-Cu composites with varying relative densities of W-Mo green compacts.

The influence of the relative density of the W-Mo green compacts on the thermal conductivity, electrical conductivity, and Vickers hardness of the W-Mo-Cu composites is illustrated in Figure 13. As shown in the figure, as the relative density of W-Mo green compacts decreases, the thermal and electrical conductivities of the composites gradually increase, reaching maximum values of 203.6 W·m^−1^·K^−1^ and 44.84 %IACS, respectively, at a relative density of 73%. The minimum values, observed at a relative density of 85%, are 171.36 W·m^−1^·K^−1^ and 29.87 %IACS, respectively. The relative density of the W-Mo green bodies significantly impacts the thermal and electrical conductivities of the composites. Given that the Cu@Gr content remains constant, the amount of carbides generated during the sintering process is essentially identical, leading to similar effects on the thermal and electrical conductivities of the composites. Therefore, this variation is primarily related to changes in Cu content within the W-Mo-Cu composites. When the relative density of the W-Mo green compact is lower, the Cu content in the composite material is higher, resulting in a complete Cu network structure that facilitates free electron transfer.

The variation in the hardness of the W-Mo-Cu composite primarily exhibits a gradual increase with an increase in the relative density of the W-Mo green compact. The minimum hardness value of 193.6 HV is observed when the relative density of the W-Mo green compact is 73%, while the maximum value of 270.56 HV is achieved at 85%. This result is primarily related to changes in Cu content within the composite material. In the W-Mo-Cu composite, the W and Mo phases possess higher hardness, whereas the Cu phase exhibits lower hardness. As the density of the W-Mo green compact gradually decreases, the composites maintain relative density within a narrow range (98.03–99.20%); however, the content of the W and Mo phases in the composite material diminishes, leading to an increase in the Cu phase content. This shift results in a downward trend in the hardness of the composite material.

In summary, the relative density of W-Mo green compacts significantly influences the densification changes of W-Mo-Cu composites both before and after sintering. Notably, the relative density of the composites remains consistently high across various relative densities of W-Mo green bodies. Furthermore, the relative density of W-Mo green compacts substantially impacts the thermal conductivity, electrical conductivity, and hardness of the composites. As the relative density of W-Mo green bodies increases, the thermal and electrical conductivities of the composites gradually decrease while their hardness increases. In practical applications, it is essential to select an appropriate relative density of W-Mo green bodies based on the specific working conditions. If higher electrical conductivity is required and strength is not a primary concern, a relatively low green compact density may be chosen; conversely, if higher strength is necessary, a relatively high green compact density should be selected.

## 4. Conclusions

In this study, Cu@Gr/W-Mo-Cu composites were fabricated through vacuum infiltration sintering at 1300 °C with a holding time of 1.5 h. The effects of Cu@Gr content (ranging from 0.2 wt.% to 1.0 wt.%) and the relative density of the W-Mo green compact (from 73% to 85%, in 3% increments) on the microstructure and properties were investigated, yielding the following findings:

(1) Without Cu@Gr, the composite contains W, Mo, and Cu phases. Upon adding 0.4 wt.% Cu@Gr, a small amount of Mo_2_C emerges, and its content increases with rising Cu@Gr content. Furthermore, the addition of Cu@Gr significantly enhances densification, reduces pores, enables uniform component distribution, and forms a Cu network between W and Mo. Moreover, composites can still maintain high densification when the Cu@Gr content exceeds 0.4 wt.%.
(2) The addition of Cu@Gr substantially improves the properties of the W-Mo-Cu composites. As the Cu@Gr content increases, both thermal and electrical conductivities and hardness significantly improve. The thermal and electrical conductivities peak at a 0.6 wt.% addition, exhibiting increases of approximately 64% and 73%, respectively, compared to the non-added case. Hardness reaches its maximum at 1 wt.%, showing a roughly 25% increase relative to the non-added state.
(3) Variations in the relative density of the W-Mo green compact have minimal effect on the phases of the composite and do not hinder the pivotal role of Cu@Gr in promoting sintering densification; composites prepared under varying relative densities remain highly dense. However, this parameter significantly influences the properties of the composite. As the relative density of the W-Mo green compact increases, the thermal and electrical conductivities of the composite decrease while its hardness increases.

## Figures and Tables

**Table 1:** Formulation of Sensitization and Activation Solution.

Chemical Reagents	Content (g/L)
SnCl_2_·2H_2_O	20.0
PdCl_2_	0.5
HCl	71.4

**Table 2:** Formulation of electroless plating solution.

Chemical Reagents	Content (g/L)
CuSO_4_·5H_2_O	10.0
Na_2_CO_3_	40.0
Na_2_EDTA	20.0
KNaC_4_H_4_O_6_·4H_2_O	40.0
C_10_H_8_N_2_	4.0
HCHO	17.7

## Data Availability

The original contributions presented in the study are included in the article, further inquiries can be directed to the corresponding author.
